# Diabetes Self-Efficacy on Glycemic Control and Well-Being of Patients With Type 2 Diabetes Mellitus: An Analytical Cross-Sectional Study

**DOI:** 10.7759/cureus.64005

**Published:** 2024-07-07

**Authors:** Aseela S, Santhi S, Anish TS, Shriraam Mahadevan

**Affiliations:** 1 Community Health Nursing, Government College of Nursing, Thiruvananthapuram, IND; 2 Nursing, Sri Ramachandra Institute of Higher Education and Research, Chennai, IND; 3 Faculty of Nursing, Sri Ramachandra Institute of Higher Education and Research, Chennai, IND; 4 Community Medicine, Government Medical College, Thiruvananthapuram, IND; 5 Endocrinology, Sri Ramachandra Institute of Higher Education and Research, Chennai, IND

**Keywords:** diabetes self-efficacy, well-being, glycemic control, hba1c, non-communicable disease clinic, t2dm

## Abstract

Introduction: Diabetes mellitus is a major, chronic, and progressive lifestyle disease. It adversely affects patients’ quality of life, effectiveness, and well-being. Self-care practices are universally recognized as imperative to keep the illness under control and prevent complications. Self-efficacy is one of the factors involved in the successful self-care of diabetic patients. The primary objective of the study was to estimate the proportion of diabetes self-efficacy and to assess the correlation of diabetes self-efficacy with glycemic control, and the well-being of patients with type 2 diabetes mellitus (T2DM). The secondary objective was to assess the factors associated with diabetes self-efficacy.

Methods: An analytical cross-sectional study was conducted among T2DM patients attending the non-communicable disease clinic in the outreach centers of Government Medical College, Thiruvananthapuram, Kerala, India. Four hundred patients with T2DM were included in the study. Diabetes self-efficacy was assessed by the Stanford Diabetes Self-Efficacy Scale and the WHO-5 index scale was used to assess wellbeing. Glycemic control was determined by HbA1C estimation, with ≤7% as good control.

Results: Among 400 patients with T2DM, 51.25 % (95% CI: 46.2-56.2) had high diabetes self-efficacy. A significantly negative correlation was found between HbA1C and self-efficacy (r =- 0.208, p = 0.01), and a positive correlation was shown between well-being and self-efficacy (r = 0.418, p = 0.01). Logistic regression analysis found that factors associated with diabetes self-efficacy were upper socioeconomic status (OR = 8.53, 95% CI: 3.06-13.82), family support (OR = 1.97, 95% CI: 1.10-3.54), participation in diabetes education classes (OR = 1.95, 95% CI: 1.10-3.54), diet compliance (OR = 4.74, 95% CI: 2.80-8.01), glycemic control (OR = 1.69, 95% CI: 1.01-2.84), and overall wellbeing (OR = 6.7, 95% CI: 3.84-11.64).

Conclusion: The proportion of high diabetes self-efficacy was 51.25% (95% CI: 46.2-56.2). The factors associated with diabetes self-efficacy were family support, participation in diabetes education classes, high socioeconomic status, absence of complications, good glycemic control, and well-being. The study findings depicted that high self-efficacy was significantly correlated with good glycemic control and well-being of patients with T2DM.

## Introduction

Diabetes is a rapidly growing health challenge that spreads across low- and middle-income nations like India. It is predicted that there will be 69.9 million diabetes cases in India by 2025, the great majority of which will remain undiagnosed [1]. Diabetes has a significant impact on individuals, their families, and the healthcare system. Due to the chronic nature of diabetes and the potential for serious complications, it leads to a substantial financial burden and a reduced quality of life [2]. Achieving favorable health outcomes requires effective daily self-management of diabetes. A sense of self-efficacy, or confidence in one's ability to govern oneself, is essential to the successful self-management of any disease [3].

Self-efficacy is a person's belief in their power to manage events or their capacity to carry out an act successfully. Self-efficacy influences treatment compliance and, thus, influences the therapeutic outcome [4]. A rise in an individual’s self-efficacy boosts their adherence to prescribed treatment for chronic illnesses. It also reflects how well a person may modify their behavior to improve their capacity for self-care. Compared to patients with low self-efficacy, those with high self-efficacy have a 20-fold increased likelihood of undergoing diabetes treatment. Self-efficacy has been applied to predict dietary habits, glycemic control, and weight management. Consequently, a better understanding of self-efficacy is integrated into planning self-management programs and promotes patient empowerment for better diabetes-related behaviors and outcomes [5].

Diabetes has a variety of effects on a person's overall health and well-being. The well-being of Individuals plays a major role in the effectiveness of diabetes management, and psychological well-being is an important goal of medical care [6]. Complications resulting from diabetes mellitus negatively affect general well-being and reduce quality of life [7]. Compared to the general population, people with T2DM have significantly lower levels of well-being. Conversely, positive well-being is a significant factor influencing the health behaviors and clinical outcomes of people with T2DM [8].

Gaining a deeper comprehension of these factors in the Indian setting can help to organize healthcare services in a way that will enhance patient outcomes and disease management. Hence, we conducted this study with the aim of understanding self-efficacy and assessing the relationship between self-efficacy and glycemic control, as well as well-being. The primary objectives of the study were to estimate the proportion of diabetes self-efficacy and assess the correlation of diabetes self-efficacy with glycemic control and the well-being of patients with T2DM. The secondary objective was to assess the factors associated with diabetes self-efficacy.

## Materials and methods

This study adopted an analytical cross-sectional research design. The study was conducted in a non-communicable disease clinic of the Integrated Family Health Centre, Pangappara, under the Medical College Health Unit, Thiruvananthapuram, Kerala, India. The period of data collection was from September 2022 to October 2023. This design was selected to estimate the proportion of diabetes self-efficacy and assess the correlation of diabetes self-efficacy with glycemic control, and the well-being of patients with T2DM. The sample size was calculated based on the previous studies, considering the primary objectives [8,9]. The sample size was fixed at 400. Approval from the Institutional Ethics Committee (IEC-NI/19/NOV/71/84) and informed consent were obtained from the participants before data collection. STrengthening the Reporting of OBservational studies in Epidemiology (STROBE) guidelines were followed for reporting the study. The inclusion criteria were both male and female adult patients with a duration of T2DM for more than one year who were willing to participate in the study. We excluded patients with known cases of depression. The sampling technique used was consecutive sampling. 

The data were collected using the interview technique. The socio-demographic data consisted of age, gender, marital status, type of family, place of residence, unhealthy habits, education, occupation, and family income. The clinical data consisted of the duration of diabetes, family history of diabetes, type of treatment for diabetes, frequency of follow-up, frequency of blood glucose test, participation in diabetes education class, presence of co-morbidity, and complications. Diabetes self-efficacy was assessed using the Stanford Diabetes Self-Efficacy Scale (DSES), an eight-item self-reported measure that evaluates participants' confidence in performing various diabetes management activities [10]. The 10-point Likert scale ranged from 1 (not at all confident) to 10 (totally confident) in performing daily diabetes activities such as diet, exercise, measures for prevention of hypoglycemia and hyperglycemia, and follow-up. The maximum total score is 80, which is converted into a percentage scale to obtain a score of 100. A percentage of ≥68.7% was considered high diabetic self-efficacy [10].

The WHO-5 index scale is used to assess well-being [11]. The WHO-5 index is a brief instrument assessing emotional well-being during the previous 2 weeks. It is a 6-point Likert scale ranging from 0 (not present) to 5 (constantly present) The scores of the items are added together and converted into a 0-100 percent scale, with higher scores indicating good well-being, with ≥50% taken as the cutoff [11]. BMI was categorized using South Asian classification. HbA1C was estimated by using the high-performance chromatographic technique, with HbA1C≤7% as good control as per Indian Council of Medical Research (ICMR) guidelines [12].

Statistical analysis

The data were analyzed using IBM SPSS Statistics for Windows, Version 20 (Released 2011; IBM Corp., Armonk, New York, United States). As the data is normally distributed, for descriptive statistical measures mean and standard deviation were used to compute the variables. A chi-square test and univariate analysis were done to find out the association between the variables of interest. Multivariate analysis was done for the significant variables in univariate analysis (p value<0.05) to find out the associated factors of Diabetes self-efficacy. Pearson's correlation coefficient was used to assess the co-relationship of diabetes self-efficacy with HbA1C and well-being. A value of p<0.05 is considered statistically significant.

## Results

A total of 400 T2DM patients participated in the study. As shown in Table 1, 55.3% of participants were females. About half of the participants (50.3%) were in the age group of 60-69 years old, and the mean age was 57.85±9.27. Considering marital status, 64% were married; 36.8% of participants had a history of unhealthy habits (smoking: 12.0%; alcoholism: 12.3%; tobacco chewing and alcoholism: 5.8%; others: 6.7%), and 79.8% of participants belonged to middle-class families (Table 1).

**Table 1:** Sociodemographic characteristics of participants

Variable		Frequency	Percentage
Age	Mean age	57.85±9.27.	
	30-39	25	6.2
	40-49	45	11.2
	50-59	122	30.5
	60-69	201	50.3
	>70	7	1.8
Gender	Male	179	44.7
	Female	221	55.3
Marital status	Unmarried	57	14.3
	Married	256	64
	Widow/widower	81	20.2
	Separated/Divorced	6	1.5
Type of family	Nuclear family	219	54.7
	Three Generation Family	160	40
	Joint family	21	5.3
Unhealthy habits	Yes	147	36.8
	No	253	63.3
Socioeconomic status	Upper	38	9.5
	Middle	319	79.8
	Lower	43	10.7

Clinical characteristics of participants

As shown in Table 2, 45.5% of participants had diabetes for more than 10 years, 54.8% had a family history of diabetes and 72.8% of were getting family support for their diabetes management, 48.5% of participants were doing regular follow-up and 42.8% were regularly checking their blood glucose. Regarding adherence to diabetic diet, 34.8% always followed diabetic diet and 52.3% participated in diabetes education programs; 80.5% of the participants had comorbidity and 36.8% had diabetes-related complications (diabetic nephropathy: 13.8%, diabetic neuropathy: 10.0%, diabetic retinopathy: 7.5%, diabetic foot: 5.5%). Only 21.8% had normal BMI; 41.2% had good glycemic control and 60.8% had good well-being.

**Table 2:** Clinical characteristics of participants T2DM: Type 2 diabetes mellitus

Variable		Frequency	Percentage
Duration of T2DM	≤10 years	218	54.5
	>10 years	182	45.5
Family history of diabetes mellitus	Yes	219	54.8
	No	181	45.2
Follow-up to healthcare agencies	Regular	194	48.5
	Irregular	205	51.5
Regularity of doing blood sugar tests	Regular	171	42.8
	Irregular	229	57.2
Family support	Yes	291	72.8
	No	109	27.2
Adhering to a diabetic diet	Always	139	34.8
	Occasionally	238	59.5
	Never	23	5.7
Diabetes education	Participated	209	52.3
	Not participated	191	47.7
Presence of comorbidity	Yes	322	80.5
	No	78	19.5
Presence of complication	Present	147	36.8
	Absent	253	63.2
Types of medications	Insulin	14	3.5
	Oral hypoglycemic agents	232	58
	Both	144	36
	Diet alone	10	2.5
BMI (based on South Asian classification)	Normal (18.5-22.9kg/m^2^)	87	21.8
	Overweight (23-24.9 kg/m^2^)	109	27.2
	Obese ( ≥25 kg/m^2^)	204	51
Glycemic control	Good (HbA1C ≤7%)	165	41.2
	Poor (HbA1C>7%)	235	58.8
Well-being	Good wellbeing ≥50%	243	60.8
	Poor wellbeing <50%	157	39.2

The variables, such as socioeconomic status (p=0.001), frequency of blood glucose test (p=0.001), family support (p=0.001), compliance to diet (p=0.001), absence of complication (p=0.03), participation in diabetes education classes (p=0.001), glycemic control (p=0.001), and well-being ((p=0.001)), had a statistically significant association with diabetes self-efficacy in univariate analysis (Table 3).

**Table 3:** Univariate analysis of sociodemographic and clinical characteristics with diabetes self-efficacy BMI: Body mass index; CI: confidence interval; DM: diabetes mellitus

Variables		Self-efficacy		χ^2^(p value)	OR(95% CI)
		High	Low		
Age	<60 yrs	49.30%	46.70%	0.271(0.61)	1.11(0.75-1.66)
	≥60 yrs	50.70%	53.30%		
Gender	Male	44.90%	44.60%	0.003(0.96)	0.99(0.67-1.47)
	Female	55.10%	55.40%		
Living status	With spouse	67.30%	60.50%	2.004(0.16)	1.34(0.89-2.02)
	Without spouse	32.70%	39.50%		
Bad health habits	Yes	38.00%	35.40%	0.31(0.58)	0.89(0.59-1.34)
	No	62.00%	64.60%		
Socioeconomic status	Upper	15.10%	3.60%	15.65(0.001)	4.87(2.08-11.37)
	Middle	74.10%	85.60%		
	Lower	10.70%	10.80%		
Duration of DM	≤10 yrs	54.10%	54.90%	0.021(0.88)	0.97(0.65-1.44)
	>10 yrs	45.90%	45.10%		
Family history of DM	Yes	53.20%	56.40%	0.42(0.52)	1.14(0.77-1.69)
	No	46.80%	43.60%		
Regularity of treatment	Regularly	68.30%	64.10%	0.799(0.37)	0.83(0.55-1.25)
	Occasionally	31.70%	35.90%		
Follow-up	Regularly	48.80%	48.20%	0.013(0.908)	0.977(0.66-1.44)
	Occasionally	51.20%	51.80%		
Blood glucose test	Regularly	50.70%	34.40%	10.95(0.001)	1.967(1.31-2.94)
	Occasionally	49.30%	65.60%		
Family support	Yes	84.90%	60.00%	31.2(0.001)	3.74(2.32-6.03)
	No	15.10%	40.00%		
Diet compliance	Regularly	54.60%	18.50%	56.09(0.001)	5.32(3,38-8.37)
	Occasionally	45.40%	81.50%		
Types of medications	Oral hypoglycemic agents	62.90%	57.90%	1.036(0.357)	0.812(0.54-1.21)
	Oral hypoglycemic agents and insulin	37.10%	42.10%		
Presence of comorbidity	Yes	77.10%	74.90%	0.266(0.345)	1.13(0.71-1.79)
	No	22.90%	25.10%		
Presence of complication	Yes	31.70%	42.10%	4.6(0.032)	1.56(1.04-2.35)
	No	68.30%	57.90%		
BMI	Normal	23.40%	20.00%	0.685(0.408)	1.22(0,76-1.97)
	Overweight and obese	76.60%	80.00%		
Diabetes education	Participated	63.40%	37.90%	25.93(0.001)	2.83(1.88-4.25)
	Not participated	36.60%	62.10%		
Glycemic control	HbA1C ≤7%	51.70%	30.30%	18.98(0.001)	1.72(1.13-2.63)
	HbA1C >7%	48.30%	69.70%		
Well-being	Good≥50	79.00%	41.50%	58.89(0.001)	5.3(3.41-8.23)
	Poor<50	21.00%	58.50%		

**Figure 1:**
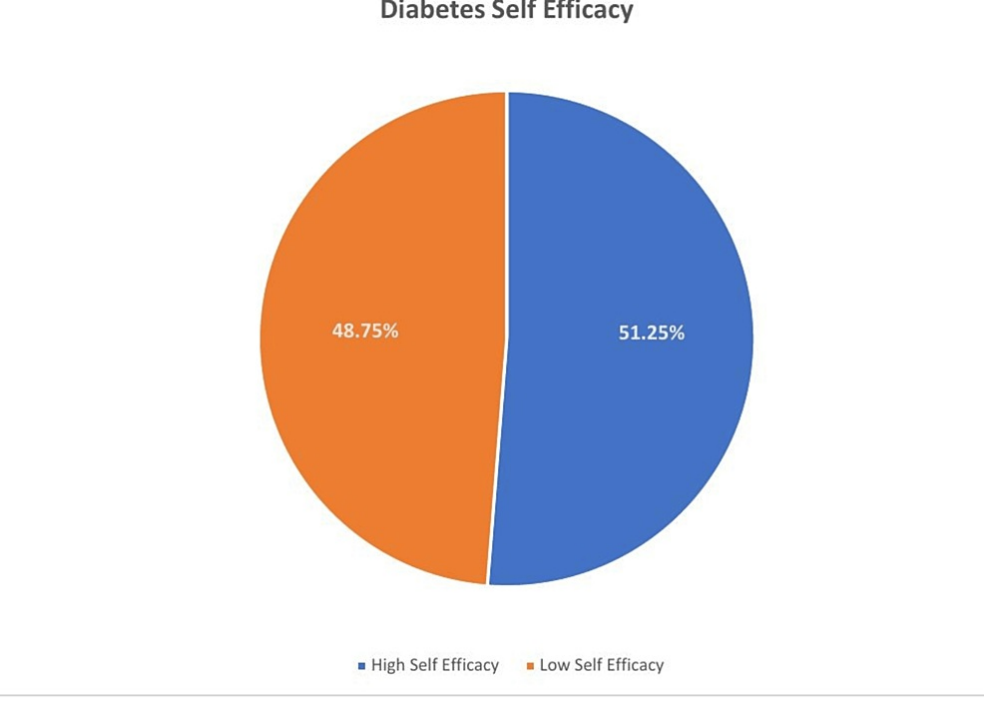
Distribution of participants based on diabetes self-efficacy

The proportion of high diabetes self-efficacy was 51.25% (95% CI: 46.2-56.2) (Figure 1).

Factors associated with diabetes self-efficacy after multivariate analysis

Table 4 shows that participants who had upper socioeconomic status were 8.53 times more likely (OR = 8.53, 95% CI: 3.06-13.82) to have high self-efficacy than those who belonged to the lower and middle classes (p value=0.001). Participants who were getting family support were 1.97 times more likely (OR = 1.97, 95% CI: 1.10-3.54) to have high self-efficacy than those who were not getting family support. Those who participated in diabetes education classes are 1.95 times more likely (OR = 1.95, 95% CI: 1.18-3.23) to have high self-efficacy than those who had not received diabetes education. Diet compliance (OR = 4.74, 95% CI: 2.80-8.01), glycemic control (OR = 1.69, 95% CI: 1.01-2.84) and well-being (OR = 6.7, 95% CI: 3.84-11.64) were the other factors associated with self-efficacy after multivariate analysis.

**Table 4:** Factors associated with diabetes self-efficacy after multivariate analysis

Variable	p-value	OR	(95% C I)
Socioeconomic status (upper class)	0.001*	8.53	3.06-13.82
Family support	0.02*	1.97	1.10-3.54
Participation in diabetes education class	0.009 *	1.95	1.18-3.23
Diet compliance (regular)	0.0001*	4.74	2.80-8.01
Blood sugar test (regular)	0.08	1.58	0.92-2.66
Absence of complication	0.17	1.44	0.86-2.41
Glycemic control	0.04*	1.69	1.01-2.84
Well-being	0.001*	6.7	3.84-11.64

Mean scores of diabetes self-efficacy, HbA1C, and well-being among participants

The mean and standard deviation of diabetes self-efficacy, HbA1C, and well-being scores were 69.81(8.55), 8.08(1.43), and 57.99(13.01), respectively (Table 5). 

**Table 5:** Mean scores of diabetes self-efficacy, HbA1C, and well-being among participants

Variable	Mean	Standard Deviation
Diabetic self-efficacy (maximum score of 100)	69.81	8.55
HbA1C	8.08	1.43
Well-being (maximum score of 100)	57.93	13.01

**Figure 2:**
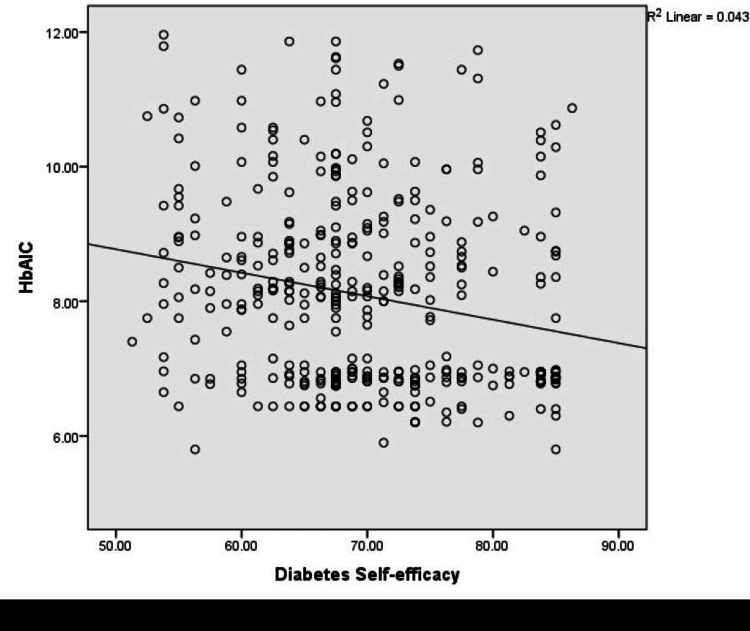
Correlation between diabetes self-efficacy and HbA1C

There is a significant negative correlation between HbA1C and diabetes self-efficacy (r =- 0.208, p =0.01) (Figure 2). 

**Figure 3:**
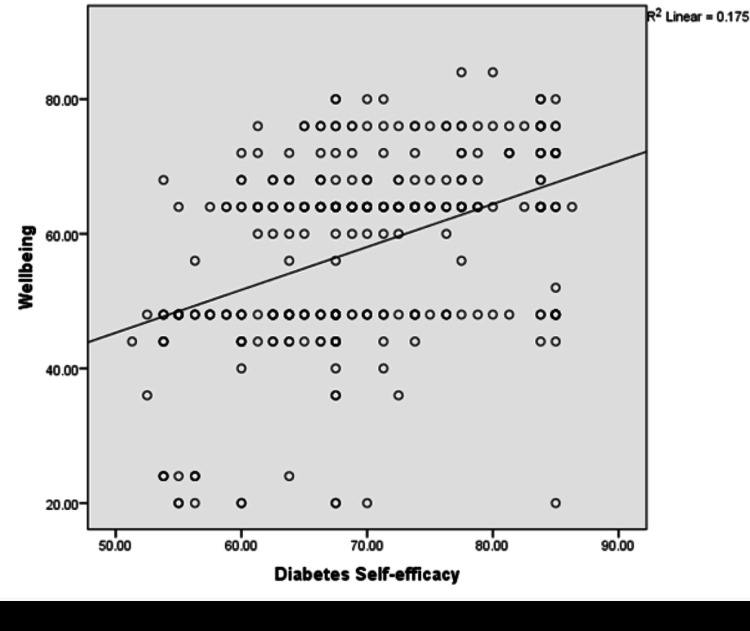
Correlation between diabetes self-efficacy and well-being

There is a significant positive correlation between well-being and diabetes self-efficacy (r = 0.418, p =0.01) (Figure 3).

## Discussion

The main purpose of this study was to estimate the proportion of diabetes self-efficacy and assess the correlation between diabetes self-efficacy, glycemic control, and well-being among patients with T2DM. In this study, 51.25% of the participants had high levels of self-efficacy. A cross-sectional survey of patients with T2DM attending Guru Tegh Bahadur Hospital, a tertiary-level hospital in Delhi, found that 42% had high self-efficacy [13]. Participants scored higher self-efficacy in a study conducted in Mexica [14]. On the other hand, the self-efficacy level is similar to that reported among patients in Nigeria, Malaysia, and Myanmar [8,9,15]. The mean score of self-efficacy was found to be 69.81±8.55 (mean ±SD), and this finding is similar among patients with T2DM attending primary care clinics run by the University of California, San Francisco [16]. The mean score for overall self-efficacy was found to be 60.61 ± 11.29 in the outpatient clinic of a university hospital in Denzil, Turkey [17] and (55.6 ± 12) in a study conducted in Thai [18].

In this study, the well-being score was 57.93±13.01 (mean ±SD). Moderate levels (69.22 ± 11.28) of well-being were present among diabetic patients in Denzil, Turkey [17]. WHO-5 psychological well-being scores below the overall mean were found in Italy, Poland, the Russian Federation, and Algeria, while the highest scores were in Mexico and Denmark [8]. Age and gender were not associated with the self-efficacy of the participants in the present study. Similar findings were seen in other studies [8,13,16,19]. Patients with higher socioeconomic status had higher self-efficacy scores, and this finding is consistent with previous studies [16,20-22]. In this study, diabetes complications were associated with low diabetes self-efficacy. The findings of the study were supported by Weaver et al., in which patients with diabetes complications were associated with lower self-efficacy [21]. However, this finding is not in agreement with a study conducted by Sharoni et al. [20]. No statistically significant association was seen between the patient’s self-efficacy and the presence of comorbidities in this study. However, a statistically significant association was found between patients with a lower score of self-efficacy and the presence of comorbidities in a study conducted in the southeast region of São Paulo state, Brazil [22]. This study found that the type of medication prescribed did not influence diabetes self-efficacy. Other studies have reported that those on insulin influence self-efficacy [13,21].

Family support was found to be a significant factor in diabetes self-efficacy in this study. Another study has documented the positive impact of supportive family members on improving the self-efficacy of people with diabetes in Thailand [18]. Patients with poorly controlled blood glucose had lower self-efficacy. This is like the findings of studies among Nigerian, Malaysian, and Thai patients, respectively [8,9,15]. A statistically significant correlation was found between patients’ diabetes self-efficacy and their well-being (r = 0.418, p = 0.01). As their self-efficacy for diabetes management increased, their well-being increased. A similar finding was seen in a study conducted in Turkey [17].

There is a significantly negative correlation between self-efficacy and HbA1C (r=- 0.208, p =0.01), and this is in agreement with a study on the effect of diabetes self-efficacy on glycemic control, medication adherence, self-care behaviors, and quality of life in a predominantly low-income, minority population were they found modest correlations between self-efficacy and glycemic control (r = −0.250, p=0.001) [23]. Some studies have found that diabetes self-efficacy does not significantly improve glycemic control [24,25]. A weak negative relationship was found between self-efficacy and HbA1c (r − 0.41, P < 0.001) among T2DM in the Malaysian primary care setting [9]. 

 A study on diabetes self-efficacy among diabetic patients attending a tertiary hospital in India demonstrated a strong positive association between self-efficacy and glycemic status, and it was the strongest factor of current glycemic status [13]. Modest correlations between self-efficacy and glycemic control (r = −0.250, p<0.001) were found among diabetic patients in low-income, minority populations in the southeastern United States [23]. This indicates that higher self-efficacy scores were significantly correlated with better glycemic control. Similar findings were demonstrated in a study amongst Turkish patients with T2DM which showed that self-efficacy had a modest negative correlation with glycemic control [26]. Another study conducted among patients with T2DM in Jordan proved that an increase in diabetes self-efficacy over time was related to an improvement in glycemic control [27]. A cross-sectional study in Myanmar has shown that patients who had a high self-efficacy level were 5.29 times more likely to have better glycemic control than patients who had an average or low self-efficacy level [15].

One of the limitations of this study is that consecutive sampling was used to recruit participants from outreach centers, which means that the results cannot be generalized to all type 2 diabetics in Kerala. Another limitation is that the study used a cross-sectional design, which precluded the deduction of causal correlations from the findings. Using self-reported questionnaires raises the risk of recall bias and social desirability bias. 

Implications for clinical practice

Assessment of self-efficacy in patients with T2DM should be a crucial initial step in developing individually customized therapies. Enhancing self-efficacy to promote better diabetes self-management should be another goal of these therapies. Measuring one's self-efficacy may be crucial to managing diabetes since it can show which patients are most likely to adhere to recommended self-care regimens. Primary care providers should work to increase their patients' sense of self-efficacy because this will help them take better care of themselves and, ultimately, glycemic control.

Future research

The barriers and facilitators affecting self-efficacy and self-care behavior should also be the subject of future research. Such evidence is necessary to guide policy change and resource allocations in the public primary care setting.

## Conclusions

The diabetes self-efficacy level of patients in this study was slightly higher than that of patients in other developing nations. Since self-efficacy is required to initiate and maintain effective self-care practices, it is an essential component of diabetes self-care practices. Measuring one's self-efficacy may be crucial to managing diabetes since it can show which patients are most likely to adhere to recommended self-care regimens. Primary care providers should work to increase their patients' sense of self-efficacy because this will help them take better care of themselves and, ultimately, glycemic control. The factors associated with diabetes self-efficacy were family support, participation in diabetes education classes, high socioeconomic status, absence of complications, good glycemic control, and well-being. A significant negative correlation was found between HbA1C and self-efficacy and a significant positive correlation was shown between wellbeing and self-efficacy.
